# Severe cerebral edema induced by watershed shift after bypass in a patient with chronic steno-occlusive disease: a case report and short literature review

**DOI:** 10.1186/s12883-020-01912-z

**Published:** 2020-09-05

**Authors:** Yin Li, Yu-yu Wei, Yang Cao, Xiao-yang Lu, Yuan Yao, Lin Wang

**Affiliations:** Department of Neurosurgery, Second Affiliated Hospital, School of Medicine, Zhejiang University, No. 88 Jiefang Rd, Hangzhou, 310009 China

**Keywords:** Cerebral edema, Bypass, Watershed shift, Magnetic resonance imaging 3D–arterial spin labeling (MRI 3D-ASL), Swollen temporal muscle

## Abstract

**Background:**

Carotid occlusive disease is a type of progressive disease resulting in ischemic stroke. Extracranial-intracranial bypass surgery represents a valid therapeutic option when medical treatment does not make effects. The appearance of cerebral edema following bypass is common during acute stage. Additionally, there are many causes of mild cerebral edema, such as hemodynamic changes, venous congestion and others. However, severe edema involving large brain tissue, which presents as reversible aphasia and hemiplegia, remains to be elucidated.

**Case presentation:**

A 55-year-old man was admitted to the neurosurgery department for repeated dizziness for over a year and sudden onset of syncope 1 month prior, and he was diagnosed with carotid occlusive disease. After surgical contraindications were excluded, dual bypass and encephalo-duro-myo-synangiosis were performed. Although blood pressure and fluid management were strictly under control promptly after surgery, massive cerebral edema involving the left anterior cerebral artery and middle cerebral artery territories occurred from the 6th day after surgery. Additionally, no discernible cerebral infarction or hemorrhage occurred. Moreover, the cerebral blood flow of the middle cerebral artery displayed an early decrease followed by delayed elevation on the left side. Without restricting the spreading of cerebral edema, life-threatening cerebral herniation could develop at any time. Mannitol and furosemide were administered for impending cerebral herniation. The amelioration of symptoms was noticed on the 16th day after surgery. The patient felt relief on the 21st day after surgery. Digital subtraction angiography performed on the 180th day after surgery demonstrated the patency of dual anastomosed vessels, and the patient recovered without any permanent neurological deficit.

**Conclusion:**

Based on changes in cerebral blood flow and reversible symptoms, the “watershed shift” phenomenon could explain such a severe deficit. However, this deficit was not the same as the classical presentation of the “watershed shift”, which involves a moderate amount of brain tissue and presents significant increases in cerebral blood flow. In addition to the “watershed shift”, a swollen temporal muscle may also participate in the progression of focal edema.

## Background

Carotid occlusive disease is a chronic cerebrovascular disease accompanied by decreased cerebral blood flow. In addition to optimal medical therapy to eliminate the poor effect of atherosclerotic plaques, surgical treatments to improve cerebral perfusion on the affected side have been performed in patients with steno-occlusive carotid artery. For patients with symptomatic nonmoyamoya cerebrovascular diseases, especially those caused by steno-occlusive carotid artery, extracranial-intracranial (EC-IC) bypass has offered no more benefits over medical therapy in the international randomized EC-IC study [1] and the Carotid Occlusion Surgery Study [2]. However, EC-IC bypass still works in certain symptomatic cerebrovascular diseases [3, 4], and neither of the two trials evaluated the effectiveness of EC-IC bypass in patients with multiple segmental stenosis, especially for those with stenotic extracranial segment of the carotid artery combined with intracranial arterial stenosis. Cerebral hyperperfusion syndrome (CHS) is a relatively rare but devastating complication of EC-IC bypass, which involves postoperative transient neurological deficits related to excessive increase in regional cerebral blood flow [5]. CHS following carotid endarterectomy (CEA) and carotid artery stenting (CAS) in patients with carotid occlusive disease has been widely reported with the evidence of hyperperfusion increasing > 100% from baseline [5], but the incidence of CHS caused by EC-IC bypass remains low [6]. “Watershed shift”, a special hemodynamic change of CHS, occurs after dual anastomosis. And the phenomenon presents more often in pediatric moyamoya disease (MMD) than in adult MMDs [7]. Here, we describe a male patient with chronic occlusion of internal carotid artery (ICA) and middle cerebral artery (MCA) who developed severe massive cerebral edema after left dual EC-IC bypass and encephalo-duro-myo-synangiosis (EDMS).

## Case presentation

**Fig. 1:**
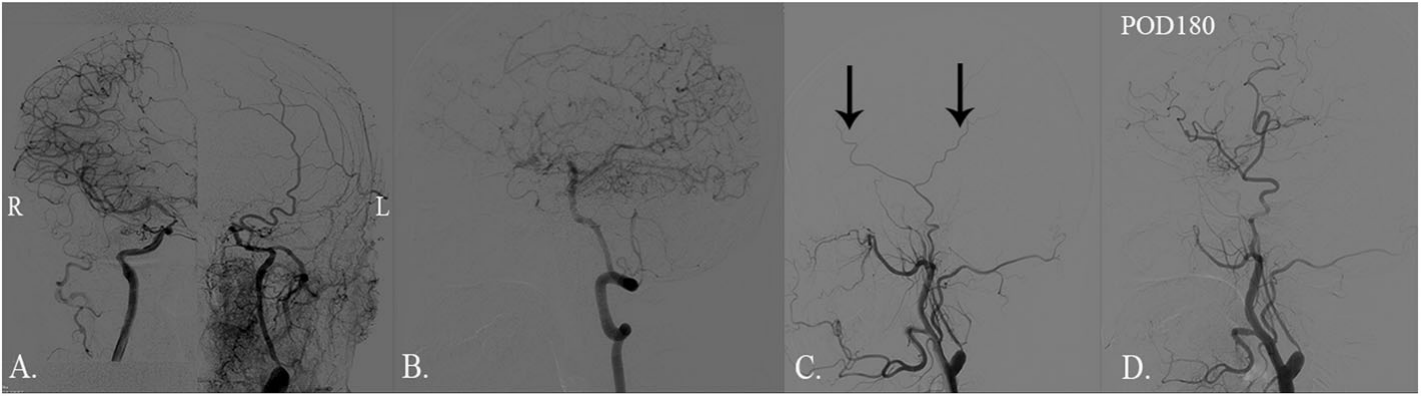
Digital subtraction angiography (DSA) results of the patient. **a**. Steno-occlusive changes at the bifurcation of the left carotid artery and abnormal development of the left ACA and MCA with a normal right ICA on preoperative DSA. **b**. Left frontal and partial lobes mainly supplied by the left posterior cerebral artery on preoperative DSA. **c**. Distribution of the superficial temporal artery before bypass surgery. The black arrows indicate the frontal and parietal branches of STA. **d**. The superficial temporal artery grew well and participated in supplying the left temporal and partial lobes in DSA examined at 6 months after surgery

**Fig. 2:**
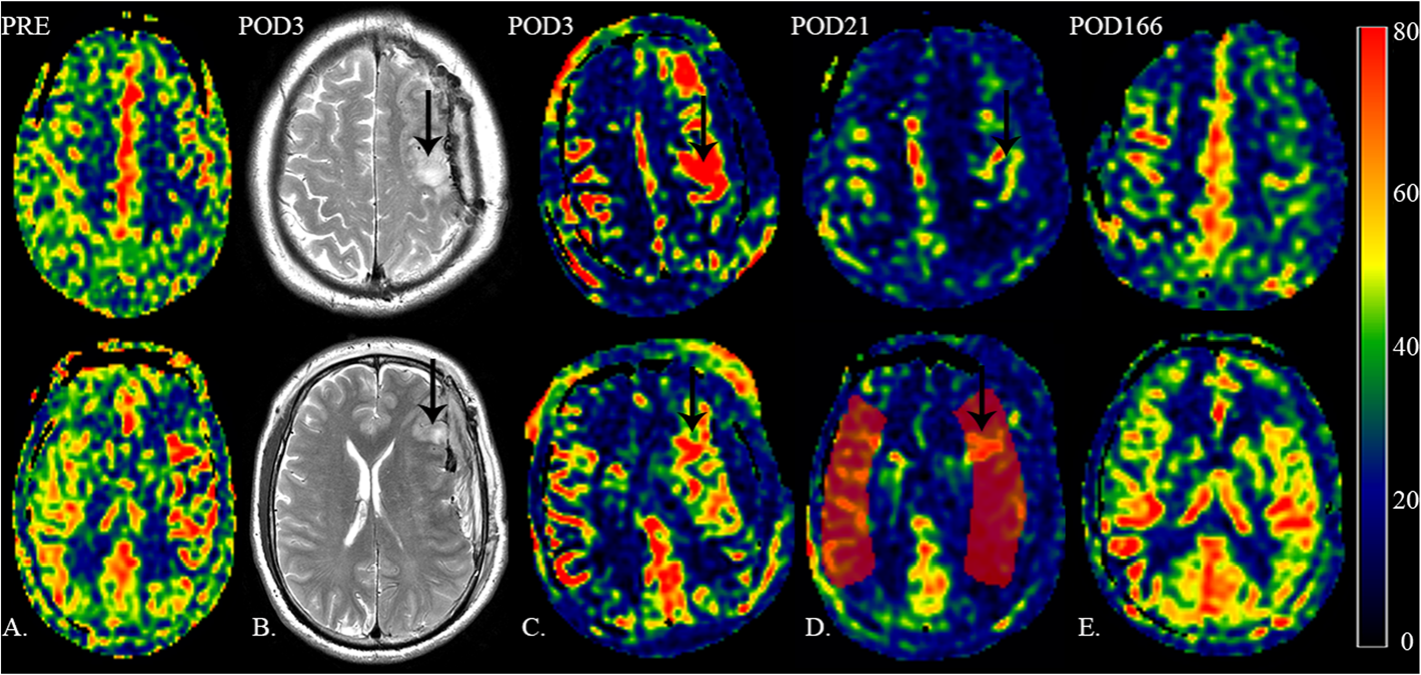
The changes of cerebral blood flow. **a**. Left cerebral perfusion is lower than right cerebral perfusion on preoperative magnetic resonance imaging 3D–arterial spin labeling (MRI 3D-ASL). **b**. T2-weighted magnetic resonance imaging showed mild focal cerebral edema in the operative area and a swollen temporal muscle on the 3rd day after surgery (The black arrow indicates cerebral edema at the sites of anastomosis). **c**. MRI 3D-ASL showed increased cerebral blood flow (CBF) at the sites of anastomosis with mildly increased CBF of the adjacent cerebral cortex on the 3rd day after surgery (The black arrow indicates increased CBF at the sites of anastomosis). **d**. MRI 3D-ASL shows high CBFs in the region distributed by dual recipient vessels compared with the adjacent cerebral cortex, while cerebral perfusion of the whole brain displays a relative low level on the 21st day after surgery (The black arrow indicates high CBFs, and the red circle indicates the region of interest of the middle cerebral artery (MCA) distribution). **e**. MRI 3D-ASL examined at 6 months after surgery shows good improvement on the left side and a normal level on the right side

**Fig. 3:**
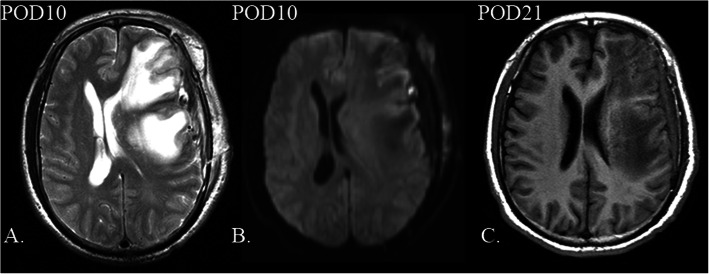
Postoperative magnetic resonance imaging (MRI) findings. **a**. and **b**. T2-weighted magnetic resonance imaging (MRI) and diffusion-weighted imaging indicate no infarction occurred on the left side, but massive cerebral edema on the 10th day after surgery. **c**. T1-weighted MRI showed mild migration of the middle line on the 21st day after surgery

A 55-year-old man who had a history of hypertension was admitted to the neurosurgery department due to dizziness repeatedly for over a year and a sudden onset of syncope 1 month prior. Additionally, he once accepted medical therapy in the neurological department without alleviation. Neurological examination revealed no abnormal signs. Diffusion-weighted imaging (DWI) showed no obvious infarct in the bilateral cerebral cortex. Ultrasound examination of the carotid artery confirmed chronic bilateral stenosis of the carotid artery bifurcation caused by stable fibrous-calcific plaques. In contrast to the normal side, left stenotic anterior cerebral arteries (ACA), middle cerebral artery (MCA) and ICA were hardly detected on preoperative DSA (Fig. 1a). The left frontal and parietal lobes were mainly supplied by the left posterior cerebral artery without obvious moyamoya vessels in skull base (Fig. 1b). Preoperative DSA confirmed the frontal and parietal branches of superficial temporal artery (STA) which originates from the external carotid artery deep to the superficial pole of the parotid and ascends anterior to the auditory canal [8] (Fig. 1c). Magnetic resonance imaging 3D–arterial spin labeling (MRI 3D-ASL) indicated decreased cerebral blood flow (CBF) in the left cerebral cortex (Fig. 2a). Dual anastomosis between the superficial temporal artery (STA) and middle cerebral artery (MCA) combined with EDMS on the left side was performed. The patency of the anastomotic stoma was immediately confirmed by indocyanine green video-angiography. Accompanied by nicardipine hydrochloride, systolic blood pressure was strictly controlled at 120–140 mmHg promptly after surgery. During the first few days, the patient presented no additional neurological deterioration. Computed tomography angiography after surgery confirmed no stenosis in recipient vessels. Additionally, T2-weighed MRI and 3D-ASL on the 3rd day after surgery showed a more significantly increased CBF at the anastomosis sites than at the preoperative stage (Fig. 2b and c), indicating the effectiveness of revascularization. Nevertheless, this patient developed aphasia and right hemiplegia on the 6th day after surgery with continuous execution of the strict program of blood pressure control. Computed tomography on the same day found that the middle line migrated to the right side and a local low-density lesion in the left frontal lobe near the operative area. Following the application of mannitol and furosemide, the symptoms began to ameliorate on the 16th day after surgery. Nevertheless, MRI 3D-ASL on the 21st day after surgery showed more decreased CBF than at the site of anastomosis on the 3rd day after surgery (Fig. 2d). T2-weighted MRI showed a massive hyperintensity lesion around the operation area, while DWI revealed no cerebral infarction, indicating massive cerebral edema in the operative area (Fig. 3a and b). Ultimately, this patient recovered after 40 days of surgery without any neurologic deficits. MRI 3D-ASL on the 166th day showed bilateral well-developed CBF (Fig. 2e) and DSA on the 180th day presented well-developed revascularization (Fig. 1d).

## Discussion and conclusions

Although the effectiveness of revascularization surgery for ischemic stroke has remained controversial in previous research [9], STA-MCA bypass is still a potential method for chronic occlusive cerebrovascular diseases, especially when medical therapy fails or neurological symptoms persist due to low cerebral perfusion. Additionally, potential postoperative complications should be addressed urgently, such as intracerebral hemorrhage, cerebral ischemic lesion or infarction resulting from “watershed shift” [10] and other deleterious complications.

Cerebral edema is defined as an increase in brain volume due to abnormal accumulation of fluid within the brain parenchyma [11], and it is classified as vasogenic, cytotoxic, hydrocephalic and osmotic edema, despite the acknowledgment that multiple types of cerebral edema are involved in most clinical situations. Regarding the mechanism, vasogenic edema is associated with micro destructions that allow increased plasma proteins and water across the blood-brain-barrier, while cytotoxic edema is accompanied by abnormal water uptake into injured brain cells [12]. Diagnostically, unlike cytotoxic edema associated with hyperintensity in DWI and decline in Apparent Diffusion Coefficient, vasogenic edema presents a variable weak change in DWI and a relative increase in water diffusion [13]. The existence of hyperintensity on T2-weighted MRI accompanied by the lack of typical hyperintensity lesions in DWI indicates that this area mainly refers to vasogenic edema (Fig. 3a and b). Postoperative cerebral edema mostly occur in MMD or those with CEA or CAS [6, 14, 15]. This condition is rare after surgery for the patients with carotid occlusive disease. The region of edema resulting from CHS is often limited to the territories near the surgical area, and hyperintensity lesions can be found in perfusion images. Among MMDs with combined bypass, retrospective analysis found that hyperperfusion-related symptoms generally occur in the first week and are entirely ameliorated during the second postoperative week [15]. Presenting neurological deficits 6 days after bypass, this patient began improving on the 17th day and completely recovered on the 40th day after surgery. The procedure to alleviate symptoms does not exactly conform to the course of postoperative CHS among MMDs. However, the dual branches of STA were anastomosed to M4 segments, making “watershed shift” possible structurally. Normalized CBF (nCBF) calculated by MRI 3D-ASL near the operative area showed an early decrease and delayed increase (Table 1) by manually choosing the region of interest in MRI 3D-ASL [16]. Meanwhile, T2-weighted MRI on the 3rd day after surgery showed early focal cerebral edema at the sites of anastomosis (Fig. 2b). In addition to transient aphasia and hemiplegia, cerebral edema and changes in perioperative nCBF can be explained by the “watershed shift”. Nevertheless, a classical “watershed shift” is defined basis on CHS, which requires ≥50% increase in the CBF at the site of anastomosis [17]. However, the absolute values of CBF on the 3rd or 21st day after surgery were both decreased below the preoperative level, even on the normal side. T1-weighted MRI on the 21st day presented mild migration of the middle line to the right side (Fig. 3c), indicating increased intracranial pressure likely still played a role in affecting cerebral perfusion in the whole brain. The steal phenomenon was thought to result in hypoperfusion on the right side. The dual bypass associated with EDMS changed the previous hemodynamics balance between the stenotic side and right side. It reduced the vascular resistance on the left side and released blood flowing into the left rather than the right hemisphere. In contrast to contralateral nCBF, we found that nCBF on the affected side increased to different degrees. Among them, the most significant difference was 1.374 vs. 0.987, which presented on the 21st day after surgery (Table 1). Different from the classical “watershed shift”, a large amount of brain tissues were involved in cerebral edema. Thus, other mechanisms may explain the progression of cerebral edema. In addition to the “watershed shift”, a swollen temporal muscle was also considered to induce focal edema. During the acute stage, a relatively narrow free bone flap could magnify the compression of the swollen temporal muscle, influencing regional cerebral circulation and resulting in local brain edema [18]. The swollen temporal muscle found on the postoperative T2-weighted MRI may exacerbate the situation. However, its effect was temporary and limited because the temporal muscle had already shrunken to normal size when the patient began to recover on the 17th day after surgery. Venous congestion was once considered as the primary cause because such massive cerebral edema associated with CHS has not been reported previously. However, venous congestion is generally related to cerebral hypoperfusion, and it seems impossible for venous congestion to be involved. Additionally, epilepsy was unrelated to severe cerebral edema in the patient in the lack of obvious symptoms of epilepsy, which has been reported as a possible cause of vasogenic edema [19]. It remains a mystery why reversible cerebral edema could lead to such severe clinical symptoms, even requiring surgical intervention. To prevent such severe cerebral edema from further into brain hernia or causing subsequent hemorrhage, one possible approach is a strict perioperative strategy. As standardized management to prevent CHS, the blood pressure should be below 130 mmHg. However, extensive low blood pressure could lead to cerebral infarction [20], and so the blood pressure of this patient was controlled under 140 mmHg by nimodipine rather than 130 mmHg. Fluid intake and hydroelectrolytic equilibration should also be considered cautiously every day. We regulated fluid intake and focused on blood tests to maintain homeostasis. Additionally, the combination of mannitol and furosemide was employed to lower intracranial pressure when clinical symptoms presented and MRI-DWI confirmed focal cerebral edema. Another possible approach is the use of edaravone, which is an antioxidant to prevent reperfusion-associated hemorrhage that can be administered to reduce effects from huge changes in CBF [21]. Additionally, quantitative magnetic resonance angiography may potentially identify patients at risk for CHS by assessing mean flow differences between ICA and MCA [22]. By applying multiple strategies, the complete disappearance of focal cerebral edema was obtained without permanent neurological deficits. Therefore, further study with a larger number of patients is necessary to validate the relationship between cerebral edema and the “watershed shift”.

**Table 1:** Cerebral perfusion calculated on MRI 3D-ASL

		PRE	POD3	POD21	POD166
L	MCA (mean ± SD) ^(a)^	40.999 ± 21.104	31.981 ± 22.397	25.802 ± 12.429	47.109 ± 18.265
	*Cerebellum* (mean ± SD) ^(a)^	29.406 ± 9.695	27.204 ± 9.943	18.782 ± 6.519	36.485 ± 8.837
	nCBF ^(b)^	1.394	1.176	1.374	1.291
R	MCA (mean ± SD) ^(a)^	44.554 ± 12.035	32.945 ± 20.146	24.473 ± 13.416	44.421 ± 16.137
	*Cerebellum* (mean ± SD) ^(a)^	39.079 ± 11.926	31.380 ± 8.041	31.380 ± 8.041	37.683 ± 12.200
	nCBF ^(b)^	1.140	1.051	0.987	1.179

*L* left, *R* right, *MCA* mean distribution of the middle cerebral artery, *Cerebellum* mean distribution of the cerebellum.^(a)^ Units: ml/100 g/min.^(b)^ nCBF means normalized CBF and nCBF = MCA (mean)/cerebellum (mean)

In conclusion, this case presents massive cerebral edema after bypass. Based on changes in cerebral blood flow and reversible symptoms, the “watershed shift” may explain this severe deficit. However, this deficit is not the same as the classical presentation resulting from the “watershed shift”, which does not involve brain tissues and presents significant increases in CBF compared with the preoperative level. In addition to the “watershed shift”, the swollen temporal muscle also participated in the progression of focal edema.

## Data Availability

The datasets used and/or analyzed during the current study are available from the corresponding author on reasonable request.

## Abbreviations

ACA: Anterior cerebral artery
CAS: Carotid artery stenting
CBF: Cerebral blood flow
CEA: Carotid endarterectomy
CHS: Cerebral hyperperfusion syndrome
DSA: Digital subtraction angiography
DWI: Diffusion-weighted imaging
EC-IC bypass: Extracranial-intracranial bypass
EDMS: Encephalo-duro-myo-synangiosis
ICA: Internal carotid artery
MCA: Middle cerebral artery
MRI 3D-ASL: Magnetic resonance imaging 3D–arterial spin labeling
STA: Superficial temporal artery

## References

[1] Group EIBS (1985). Failure of extracranial-intracranial arterial bypass to reduce the risk of ischemic stroke. Results of an international randomized trial. N Engl J Med.

[2] Powers WJ, Clarke WR, Grubb RL, Videen TO, Adams HP, Derdeyn CP (2011). Extracranial-intracranial bypass surgery for stroke prevention in hemodynamic cerebral ischemia: the carotid occlusion surgery study randomized trial. JAMA..

[3] Low SW, Teo K, Lwin S, Yeo LL, Paliwal PR, Ahmad A (2015). Improvement in cerebral hemodynamic parameters and outcomes after superficial temporal artery-middle cerebral artery bypass in patients with severe stenoocclusive disease of the intracranial internal carotid or middle cerebral arteries. J Neurosurg.

[4] Muroi C, Khan N, Bellut D, Fujioka M, Yonekawa Y (2011). Extracranial-intracranial bypass in atherosclerotic cerebrovascular disease: report of a single Centre experience. Br J Neurosurg.

[5] van Mook WN, Rennenberg RJ, Schurink GW, van Oostenbrugge RJ, Mess WH, Hofman PA (2005). Cerebral hyperperfusion syndrome. Lancet Neurol.

[6] Yamaguchi K, Kawamata T, Kawashima A, Hori T, Okada Y (2010). Incidence and predictive factors of cerebral hyperperfusion after extracranial-intracranial bypass for occlusive cerebrovascular diseases. Neurosurgery..

[7] Yu J, Hu M, Yi L, Zhou K, Zhang J, Chen J (2019). Paradoxical association of symptomatic cerebral edema with local hypoperfusion caused by the 'watershed shift' after revascularization surgery for adult moyamoya disease: a case report. Ther Adv Neurol Disord.

[8] Hou K, Guo Y, Xu K, Yu J (2019). Clinical importance of the superficial temporal artery in neurovascular diseases: a PRISMA-compliant systematic review. Int J Med Sci.

[9] Lee JI, Jander S, Oberhuber A, Schelzig H, Hanggi D, Turowski B (2014). Stroke in patients with occlusion of the internal carotid artery: options for treatment. Expert Rev Neurother.

[10] Tu XK, Fujimura M, Rashad S, Mugikura S, Sakata H, Niizuma K (2017). Uneven cerebral hemodynamic change as a cause of neurological deterioration in the acute stage after direct revascularization for moyamoya disease: cerebral hyperperfusion and remote ischemia caused by the 'watershed shift'. Neurosurg Rev.

[11] Nag S, Manias JL, Stewart DJ (2009). Pathology and new players in the pathogenesis of brain edema. Acta Neuropathol.

[12] Sakata H, Fujimura M, Mugikura S, Sato K, Tominaga T (2015). Local Vasogenic edema without cerebral Hyperperfusion after direct revascularization surgery for Moyamoya disease. J Stroke Cerebrovasc Dis.

[13] Karapanayiotides T, Meuli R, Devuyst G, Piechowski-Jozwiak B, Dewarrat A, Ruchat P (2005). Postcarotid endarterectomy hyperperfusion or reperfusion syndrome. Stroke..

[14] Chan PH (1996). Role of oxidants in ischemic brain damage. Stroke..

[15] Hayashi K, Horie N, Suyama K, Nagata I (2012). Incidence and clinical features of symptomatic cerebral hyperperfusion syndrome after vascular reconstruction. World Neurosurg.

[16] Ha JY, Choi YH, Lee S, Cho YJ, Cheon JE, Kim IO (2019). Arterial spin labeling MRI for quantitative assessment of cerebral perfusion before and after cerebral revascularization in children with Moyamoya disease. Korean J Radiol.

[17] Tashiro R, Fujimura M, Kameyama M, Mugikura S, Endo H, Takeuchi Y (2019). Incidence and risk factors of the watershed shift phenomenon after superficial temporal artery-middle cerebral artery anastomosis for adult Moyamoya disease. Cerebrovasc Dis.

[18] Fujimura M, Kaneta T, Shimizu H, Tominaga T (2009). Cerebral ischemia owing to compression of the brain by swollen temporal muscle used for encephalo-myo-synangiosis in moyamoya disease. Neurosurg Rev.

[19] Hong KS, Cho YJ, Lee SK, Jeong SW, Kim WK, Oh EJ (2004). Diffusion changes suggesting predominant vasogenic oedema during partial status epilepticus. Seizure..

[20] Kronenburg A, Braun KP, van der Zwan A, Klijn CJ (2014). Recent advances in moyamoya disease: pathophysiology and treatment. Curr Neurol Neurosci Rep.

[21] Uchino H, Nakayama N, Kazumata K, Kuroda S, Houkin K (2016). Edaravone reduces Hyperperfusion-related neurological deficits in adult Moyamoya disease: historical control study. Stroke..

[22] Andereggen L, Amin-Hanjani S, El-Koussy M, Verma RK, Yuki K, Schoeni D (2018). Quantitative magnetic resonance angiography as a potential predictor for cerebral hyperperfusion syndrome: a preliminary study. J Neurosurg.

